# Supplementary material for: Meso-scale seabed quantification with geoacoustic inversion
Source: Commun Eng. 2024 Apr 3;3:60. doi: 10.1038/s44172-024-00204-5 (PMC10991265; doi:10.1038/s44172-024-00204-5)
Supplement: Supplementary file 2 — Supplemental Information [file 44172_2024_204_MOESM2_ESM.pdf]

Supplementary Material:
Meso-scale seabed quantification with geoacoustic inversion

Tim Sonnemann[1,2], Jan Dettmer[1], Charles W. Holland[2], and Stan E. Dosso[3]

[1]Department of Geoscience, University of Calgary, Calgary AB Canada
[2]Portland State University, Portland OR, USA
[3]School of Earth and Ocean Sciences, University of Victoria, Victoria BC Canada

February 9, 2024

Contents

List of Figures **2**

List of Tables **2**

1 Supplementary Note 1 **2**

2 Supplementary Figures **3**

3 Supplementary Tables **7**

List of Figures

1 **Posterior probability densities (PPDs) of data misfit and number of sediment layers.** Gray scale heatmaps of marginal PPDs along track position for: (a) Root-mean-squared (RMS) reflection coefficient (RC) misfit, and (b) number of layers. 3
2 **95% credibility interval (CI) width for inferred geoacoustic parameters.** Porosity, compressional-wave grain shearing modulus γ_{p}, material exponent n, sound speed c_{p}, density ρ, and attenuation α_{p} (top to bottom). Note that the uncertainty generally increases with depth which is expected in this inversion. The layer interfaces are thin zones of increased CI width due to depth uncertainty. 4
3 **Median of posterior probability densities of all geoacoustic parameters.** Porosity, compressional-wave grain shearing modulus γ_{p}, material exponent n, sound speed c_{p}, density ρ, and attenuation α_{p} (top to bottom). The track bathymetry is shown with depth relative to the sea surface. The main report omits the grain shearing modulus and material exponent. 5
4 **Characterization of reflection coefficient data artifact.** (a) Original reflection coefficient values of one ping (11 averaged pings centered on ping 12 at 1113 Hz). (b) The same data as in (a) corrected for the noted sawtooth pattern. (c) Fifth-order polynomials fitted at each frequency to the averaged reflection coefficients of all pings. (d) Difference between the fitted polynomial values and the mean reflection coefficients of all pings, resulting in a fine oscillatory pattern. 6

List of Tables

1 Uniform distribution parameters of prior densities. 7
2 Upper and lower linear section boundaries of the $P(\beta|\gamma_{\mathrm{p}})$ prior distribution. 7
3 Upper and lower curve coefficients of the $P(c_{\mathrm{p}}|\rho)$ prior distribution. 7

Supplementary Note 1

In any inversion, it is crucial to examine the assumptions and the details of the inversion statistics. A measure for the inversion misfit is the root-mean-square (RMS) residual between predicted and observed data, which is plotted for all data sets along the track using 5000 random samples per data set in Fig. 1a. The mean RMS misfit of 0.03 corresponds to about 8 to 30% of reflection coefficient values from 0.1 to 0.4, respectively. The mean of all data sets' standard error estimates is 0.058, while the RMS misfit values are largely below that estimated data error, which might indicate some degree of over-fitting. The few spikes of higher RMS values along track result from lower quality data which were fitted less well by the inversion.

The number of inferred layers is mostly between 6 to 12, as shown for the track in Fig. 1b. Regions with somewhat higher misfit and increased number of inferred layers are associated with channel-like near-surface structures. These appear above concave folded or strongly faulted bedding of the erosional layer and the channels could be artifacts due to wave-field focussing in these areas which would be a violation of our 1D model assumption.

The 95% credibility intervals widths of all parameters along the track indicate increasing uncertainty with depth (Fig. 2). In particular, the half-space is not well constrained, and some pings did not converge to a well resolved solution such as those close to 8.2 km and 9.6 km along-track position.

Supplementary Figures

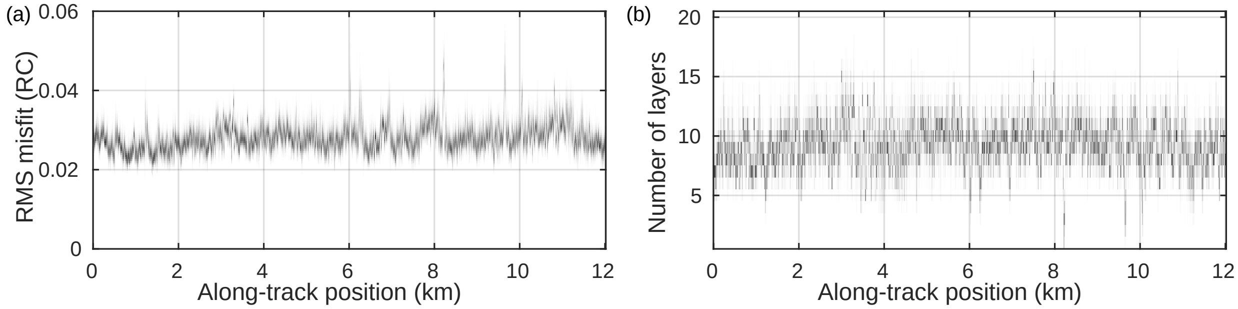

Supplementary Figure 1: Posterior probability densities (PPDs) of data misfit and number of sediment layers. Gray scale heatmaps of marginal PPDs along track position for: (a) Root-mean-squared (RMS) reflection coefficient (RC) misfit, and (b) number of layers.

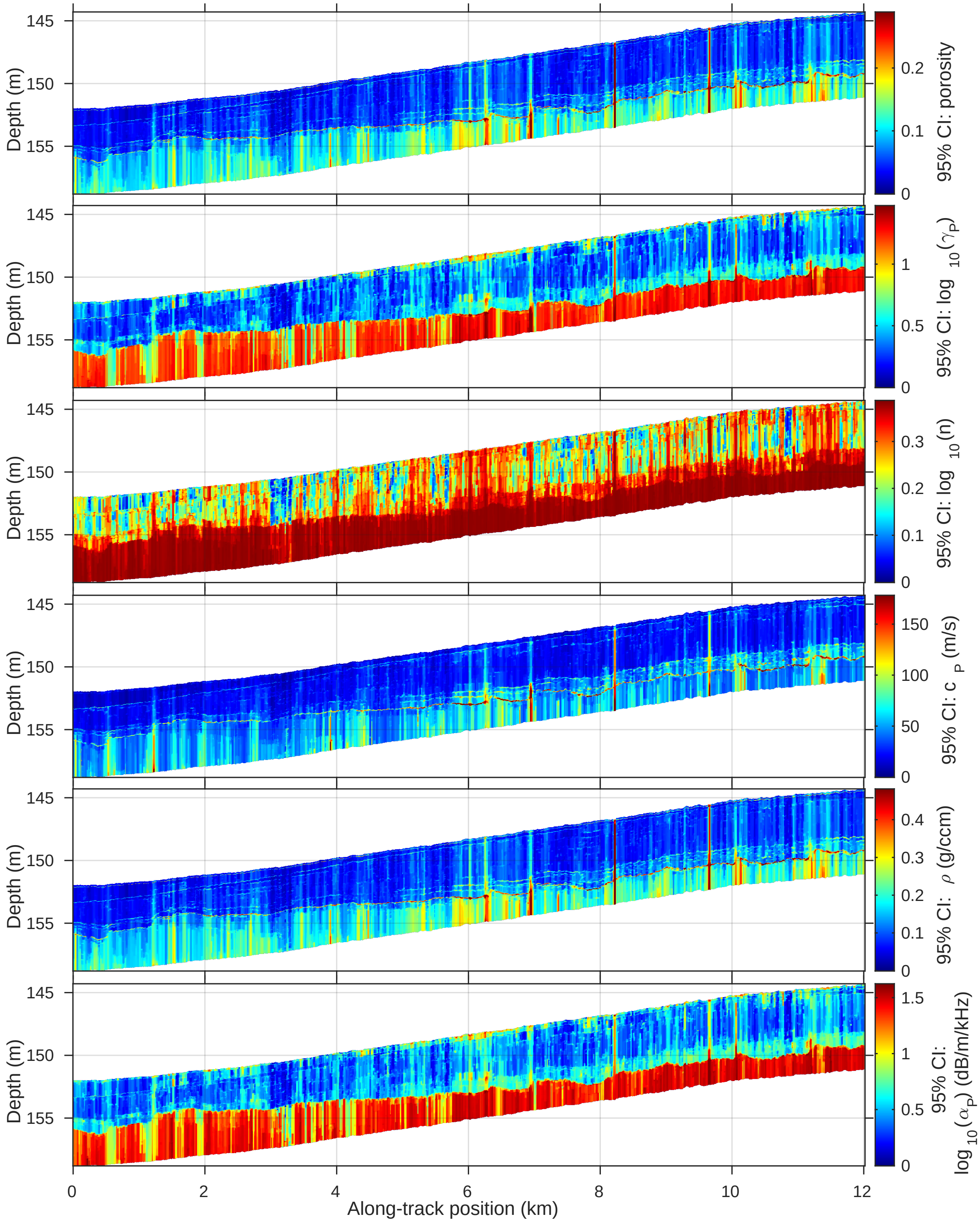

Supplementary Figure 2: 95% credibility interval (CI) width for inferred geoacoustic parameters. Porosity, compressional-wave grain shearing modulus γ_p, material exponent n, sound speed c_p, density ρ, and attenuation α_p (top to bottom). Note that the uncertainty generally increases with depth which is expected in this inversion. The layer interfaces are thin zones of increased CI width due to depth uncertainty.

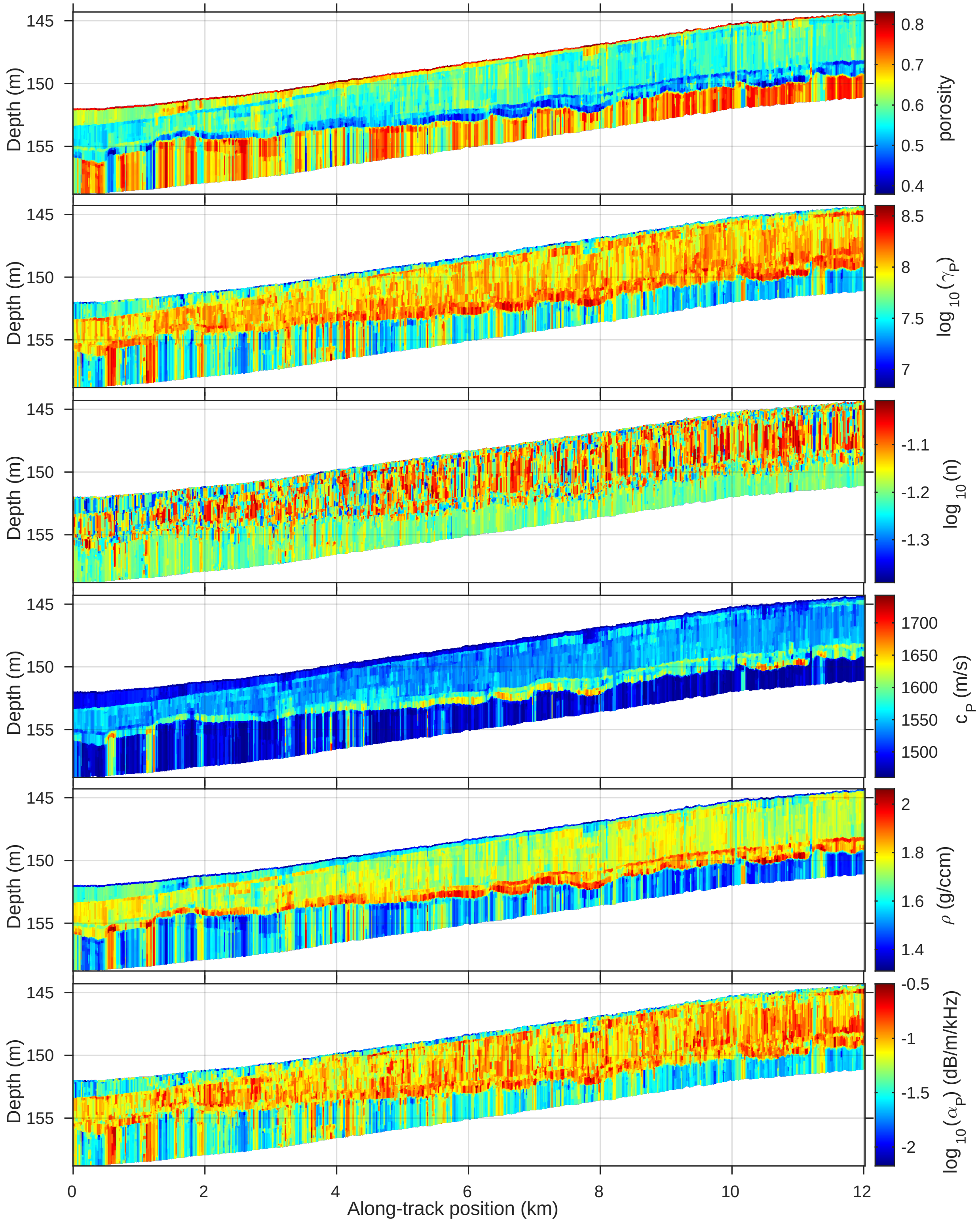

Supplementary Figure 3: Median of posterior probability densities of all geoacoustic parameters. Porosity, compressional-wave grain shearing modulus γ_{p}, material exponent n, sound speed c_{p}, density ρ, and attenuation α_{p} (top to bottom). The track bathymetry is shown with depth relative to the sea surface. The main report omits the grain shearing modulus and material exponent.

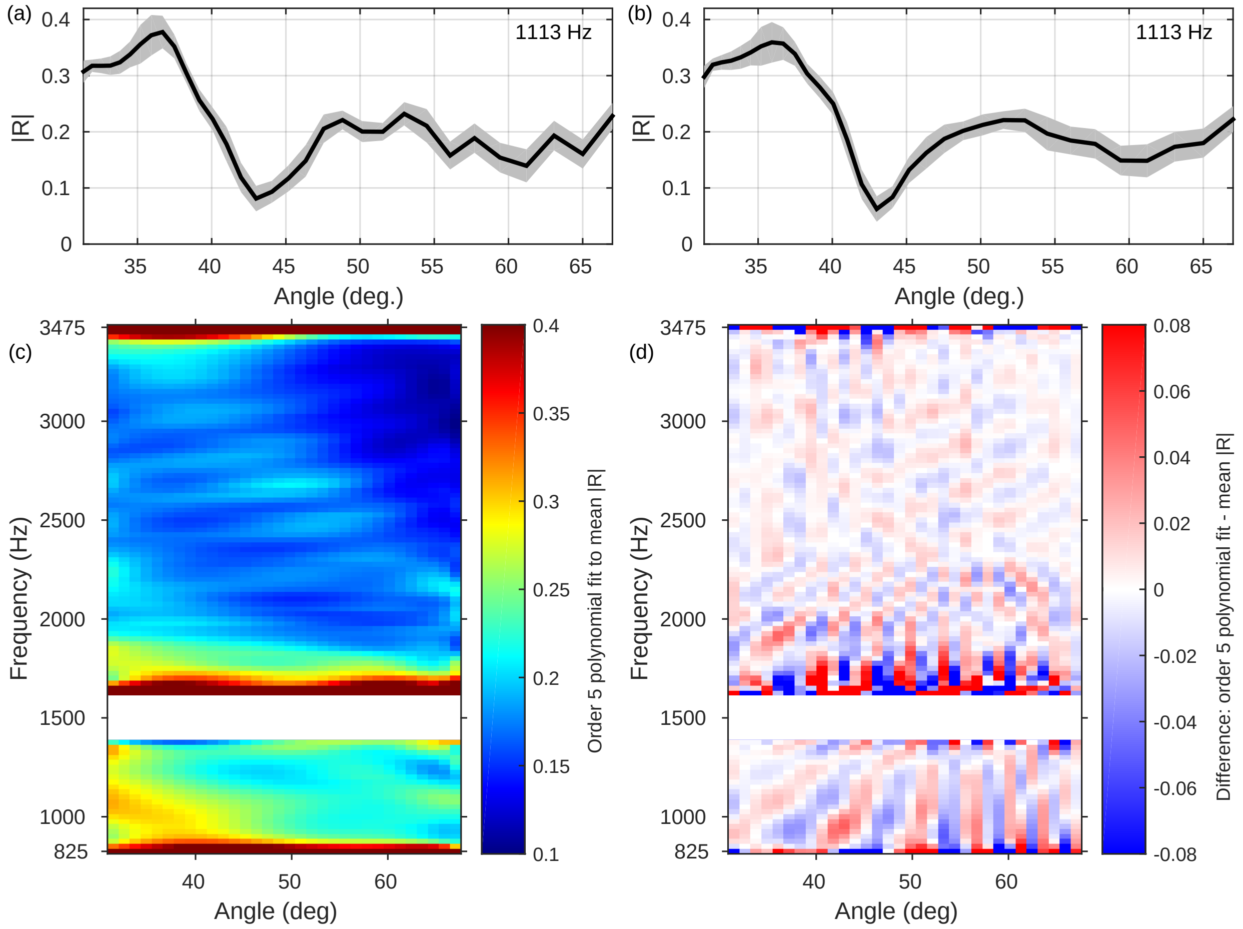

Supplementary Figure 4: Characterization of reflection coefficient data artifact. (a) Original reflection coefficient values of one ping (11 averaged pings centered on ping 12 at 1113 Hz). (b) The same data as in (a) corrected for the noted sawtooth pattern. (c) Fifth-order polynomials fitted at each frequency to the averaged reflection coefficients of all pings. (d) Difference between the fitted polynomial values and the mean reflection coefficients of all pings, resulting in a fine oscillatory pattern.

Supplementary Tables

Setting the prior information is important in any inverse problem. The priors can be informed by theoretical or empirical limits. In general, our approach was to keep the priors as wide as possible to allow the data, not the priors to determine the parameters. These prior bounds encompass all marine unconsolidated sediments. The bounded parameters are porosity β, compressional-wave grain shearing modulus γ_{p}, material exponent n, sound speed c_{p}, and density ρ.

Supplementary Table 1: Uniform distribution parameters of prior densities.

Parameter	Lower bound	Upper bound
Number of layers	1	20
Interface depth	0.05 m	6.8 m
$\log_{10}\gamma_{\mathrm{p}}$	6.4	9.4
$\log_{10} n$	-1.4	-1.0
β (0.0 m)	0.2	1.0
β (4.0 m)	0.0	0.8
β (6.8 m)	0.0	0.8
ρ	1.25 g/cm^3	2.25 g/cm^3

Supplementary Table 2: Upper and lower linear section boundaries of the $P(\beta|\gamma_{\mathrm{p}})$ prior distribution.

β	0	0.15	0.3	0.6	0.85	1.0
$(\gamma_{\mathrm{p}})_{\mathrm{l}}$ [Pa]	$2\cdot10^{10}$	$3.55\cdot10^{9}$	$3.5\cdot10^{8}$	$1\cdot10^{7}$	$2.49\cdot10^{6}$	$2.2\cdot10^{6}$
$(\gamma_{\mathrm{p}})_{\mathrm{u}}$ [Pa]	$4\cdot10^{10}$	$7.6\cdot10^{9}$	$1.8\cdot10^{9}$	$2.25\cdot10^{8}$	$6.2\cdot10^{7}$	$1.8\cdot10^{7}$

Supplementary Table 3: Upper and lower curve coefficients of the $P(c_{\mathrm{p}}|\rho)$ prior distribution.

Curve	g_1	g_2	g_3	g_4	g_5
Lower	1.515	0.890	0.3695	1.87	1.5004
Upper	1.580	0.907	0.3695	2.05	1.5014
